# Exploring experiences of forced clearances in people with hoarding difficulties

**DOI:** 10.1111/papt.70082

**Published:** 2026-06-07

**Authors:** Hannah Parker, Louise Waddington, James D. Gregory

**Affiliations:** ^1^ School of Psychology Cardiff University Cardiff UK

**Keywords:** forced clearance, hoarding disorder, qualitative, thematic analysis, trauma

## Abstract

**Introduction:**

People with Hoarding Disorder (PWHD) can experience enforced removal of their possessions by others, often with the intention of trying to improve an individual's situation. To date, there has been no research exploring PWHD's experiences of this. The study qualitatively explored people's experiences of forced clearances within the context of HD.

**Methods:**

Nine participants meeting DSM‐5 criteria for HD completed a semi‐structured interview about their experience of forced clearances; this included a description of the clearance, impact upon self and hoarding behaviours and recommendations on what improved support could look like. Participants also completed questionnaires measuring hoarding severity and distress caused by a forced clearance.

**Results:**

Three themes and six subthemes were generated: (1) Power Imbalance (subthemes: Criminalisation of Mental Health Difficulties; Clearance as the Lesser of Two Evils); (2) The Trauma of Clearance (subthemes: Losing Belongings is Like Losing Myself; ‘Trauma on top of Trauma’); (3) Picking Up the Pieces (subthemes: Clearance Weighs Heavily on the Mind; Replacing the Shield). Participant recommendations included the importance of early intervention, the need to build trust and seeing the person behind the belongings. Most scores on the measure of distress were high, consistent with participant reports that the event was typically a traumatic experience.

**Conclusion:**

Findings suggest that forced clearances are a traumatic experience with a long‐lasting impact for PWHD. The study has theoretical implications for how traumatic events are defined in the context of HD and clinical implications for how to support PWHD and their experience of forced clearances.

## INTRODUCTION

Hoarding Disorder (HD) is defined as “persistent difficulty discarding or parting with possessions”, which impacts upon areas of functioning such as social activities and occupation (American Psychological Association [APA], 2013). The prevalence of HD is estimated at 2.5% (Postlethwaite et al., 2019). HD has a significant impact on individual functioning (Ong et al., 2015); people with HD (PWHD) score lower on measures of quality of life, experience high rates of unemployment and are more likely to be the victims of crime (Saxena et al., 2011). This group also reports increased loneliness and feels less socially supported when compared to people with Obsessive Compulsive Disorder (OCD; Edwards et al., 2023). Functional impairment is just as elevated in relatives of those with HD as in the individual themselves, with the carer burden equivalent to or higher compared to relatives of those with dementia (Drury et al., 2014). There are also impacts on the safety of the home, with concerns about environmental health and fire risk being prevalent (Porter & Hanson, 2022). Hoarding difficulties also have costly economic outcomes for housing providers and emergency services (Neave et al., 2017).

The complexity of HD is reflected in the approaches and outcomes of the interventions that people can receive, with hoarding severity often determining the extent of intervention required. For example, PWHD with a certain level of insight and motivation to address their difficulties may be offered Cognitive Behavioural Therapy (CBT) for HD, which focusses on reducing acquisition and increasing discarding of possessions (Steketee et al., 2010); however, outcomes show that after receiving an average of 26 sessions (with sessions often being multiple hours in duration) over approximately a year, only 24% of participants are considered to be “very much improved” (Tolin et al., 2015). In more severe cases, a multidisciplinary intervention may be required involving input from agencies including mental health, social work, housing and environmental health, amongst others. If an agency judges that the condition of a property poses a significant risk to individuals inside and/or outside of the home, then an enforced intervention may be deemed necessary, which can include a forced clearance. Forced clearance interventions may take place as a result of the application of statutory powers, such as Section 83 of the Public Health Act (1936) in England and Wales, whereby a property is deemed “filthy or unwholesome so as to be prejudicial to health” or if vermin are present. It may also be necessary to investigate safeguarding issues, with hoarding in England considered a self‐neglect concern under the Care Act (2014), and to potentially assess capacity under the Mental Health Act (1983). Clearances can also occur in other contexts, for example, by family and friends or non‐statutory services; however, little is known about this. Social housing landlords have described the challenge of attempting to manage hoarding difficulties in a person‐centred way, whilst also recognising that enforcement action such as clearance of the property or eviction may be required, despite being aware of the resulting harm this may cause (Porter & Hanson, 2022). PWHD have deemed cleaning and removal services to be one of the least acceptable treatment options (Rodriguez et al., 2016); this type of intervention is therefore resisted by PWHD and they are avoidant of support which exerts pressure to discard (McGrath et al., 2024). Given this, a forced clearance is a significant stressful life event for PWHD. Stressful life events, specifically involving the loss of possessions, play a significant role in HD, with nearly a third reporting having had something taken by force (Hartl et al., 2005) and 61% having reported loss or damage to possessions (Tolin et al., 2010). Forced clearances may be distressing for PWHD because they remove the feeling of security and safety that possessions provide them with. The Site‐Security Model (Kellett, 2007) offers an evolutionary understanding of hoarding in which resources are gathered and stored at the individual's “site”, usually their home, providing psychological safety. A similar response can be observed in non‐clinical populations; for example, during the Covid‐19 pandemic, resources were stockpiled, or hoarded, during a time of resource unavailability. The keeping of objects provides a sense of safety and security for PWHD in several ways. For example, some individuals with HD have such a heightened emotional attachment to their belongings because they are perceived as extensions of themselves and/or others (Frost et al., 1995; Kings et al., 2017, 2021). Other possession beliefs include *material scrupulosity, that is, an exaggerated responsibility to prevent waste* (Frost et al., 2018); objects offering stability and a form of identity expression (Tinlin et al., 2022); and objects as prompts for memory (Steketee et al., 2003). Further, emotional attachment and responsibility belief types are strongly associated with difficulty discarding in HD (Smith et al., 2026). Taken together, this evidence demonstrates what makes possessions important to PWHD, and therefore the potential significant distress caused by an enforced loss of possessions.

In addition to causing distress, forced clearances may have the unintended consequence of worsening hoarding behaviour, which is unhelpful for both individuals and services. Recent research has demonstrated object attachment as a mediator between loneliness and symptoms of HD (Yap et al., 2020); with hoarding as a compensatory behaviour for unmet interpersonal needs, a clearance of belongings could therefore serve to strengthen object attachment. Clearances are reported to not result in long‐lasting improvement, and hoarding persists (Frost et al., 2003). Furthermore, the experience of adverse life events in PWHD is related to increased hoarding severity (Chia et al., 2021), and consequently the stress of a forced clearance may contribute to hoarding behaviour and lack of engagement with treatment in the future (Gibson et al., 2010). Events related to loss can also spur hoarding motivated by memory concerns (Fontenelle et al., 2021).

Professionals can find it challenging to support PWHD and face difficult decisions regarding care (Parker et al., 2025); recent research showed that a large proportion of surveyed professionals endorsed a coercive method of intervention (Larkin et al., 2025). To date, PWHD's experiences of forced clearances and the degree to which they are experienced as traumatic, or not, has not been investigated. Seeking the perspectives of PWHD is important for numerous reasons, including helping those involved in supporting PWHD to understand the impact of forced clearances and therefore inform intervention decisions and their delivery. The present study intended to answer the following research question: What are people's experiences of forced clearances within the context of HD?

## MATERIALS AND METHODS

### Design

The study was given ethics approval by the School of Psychology Research Ethics Committee (SREC number: EC.23.06.13.6806RA). The study protocol was pre‐registered with the Open Science Framework: https://osf.io/ds7ug/overview. The study used a convergent parallel research design (Cresswell & Plano Clark, 2011), consisting of semi‐structured interviews to collect qualitative data, which was analysed using Reflexive Thematic Analysis (Reflexive TA; Braun & Clarke, 2006, 2021), and questionnaires to gather quantitative data. Reflexive TA is a methodology suitable for understanding experiences and shared meaning across the participant sample (Braun & Clarke, 2022). The questionnaire data gathered allowed the participants' experience to be quantitatively measured in relation to trauma; this provided the opportunity for converging evidence regarding the traumatic nature of enforced clearance, should this emerge as a theme from the qualitative data.

### Participants

Participants were required to meet inclusion criteria of being aged 18 years or above and UK‐based. Questions were asked to establish at the earliest stage whether participants were likely to fulfil DSM‐5 (APA, 2013) criteria for HD. Therefore, to be eligible to participate, people also had to answer yes to the following questions taken from the Structured Interview for Hoarding Disorder (SIHD; Nordsletten et al., 2013): “Do you experience difficulty discarding or parting with possessions? This may include throwing away, selling, giving away, recycling, etc.”; “Do you have any general medical conditions, a history of head injury or cerebrovascular disease?”, and if applicable: “Did you have difficulties with discarding/clutter before you became ill?”. Finally, participants also had to report that they had experienced one or more instances of forced clearance of their possessions. The emphasis was on the clearance being experienced as forced; there were no restrictions on the number of items or who imposed the forced clearance, which may have been undertaken by organisations and people including, but not limited to, local authorities, housing associations, and family and friends.

Nine participants were recruited via hoarding support groups, charities such as Hoarding UK, social media channels of hoarding organisations, and other third‐party agencies working with people with hoarding difficulties. Participants were reimbursed with a £20 gift voucher. The difficulties in selecting a sample size number for qualitative research are well documented, with Reflexive TA moving away from the concept of selecting a sample size to satisfy data saturation (Braun & Clarke, 2021). Instead, the information power model (Malterud et al., 2016) suggests that specific study aims in a well‐defined target population would indicate higher information power and therefore require fewer participants, which is relevant to the population recruited in the present study.

### Measures

#### The Structured Interview for Hoarding Disorder (SIHD)

The SIHD (Nordsletten et al., 2013) was administered to confirm participants met criteria for HD. The tool was developed in line with DSM‐5 (APA, 2013) criteria, with HD representing a new addition to the most recent DSM. The underlying criteria have excellent inter‐rater reliability and are able to differentiate between normative collecting, sub‐clinical hoarding and HD, with the diagnosis deemed acceptable by PWHD (Mataix‐Cols et al., 2013).

#### The Saving Inventory‐Revised (SI‐R)

The SI‐R (Frost et al., 2004) is a 23‐item self‐report measure used to assess hoarding severity across three subscales: Clutter; Difficulty Discarding; and Excessive Acquisition. The SI‐R has high discriminant validity and excellent internal reliability (Cronbach's *α* = 0.93; Kellman‐McFarlane et al., 2019). Items are scored from 0 to 4, with higher scores representing greater hoarding severity. A score of 40 or higher is indicative of clinically significant hoarding.

#### The Impact of Events Scale‐Revised (IES‐R)

The IES‐R (Weiss & Marmar, 1996) is a 22‐item self‐report measure used to gauge current distress resulting from a stressful life event; therefore, participants were asked to answer in relation to their experience of a forced clearance. The IES‐R has good psychometric properties (Weiss, 2007), including internal reliability (Sundin & Horowitz, 2002). Items are scored 0 to 4, with higher scores indicating greater distress. A score of 33 or higher represents probable PTSD.

### Semi‐structured interview

A semi‐structured interview schedule was created to investigate experiences of forced clearances. This was developed using guidance from Magnusson and Marecek (2015) and covered the following areas: the nature of the clearance; how it was perceived; how unhelpful/helpful the clearance was; what emotions were experienced in relation to the clearance; what impact the clearance had; any support received; how they coped; impact on relationships; and impact on HD. To increase the likelihood that the experience was emotionally salient, participants were interviewed in relation to their most prominent experience of forced clearance, but were offered the opportunity to talk about up to two experiences. Opportunities for participants to ask questions, wellbeing checks and time for breaks were designed into the schedule. A person with personal experience of hoarding difficulties who had experienced a forced clearance of their own possessions reviewed a draft of the interview schedule and provided feedback regarding the structure and content of the interview. The service user was reimbursed for their time as per National Institute for Health and Care Research (NIHR) guidelines (NIHR, 2022).

### Procedure

Potential participants were provided with a Participant Information Sheet and Consent Form and screened for eligibility either via email or on the telephone. Participants meeting the inclusion criteria were then invited to arrange an interview. After providing consent, participants first answered brief demographic questions (age, gender, ethnicity) before completing the SIHD (Nordsletten et al., 2013) followed by the SI‐R (Frost et al., 2004) to assess hoarding severity. This was followed by the semi‐structured interview and then the IES‐R (Weiss & Marmar, 1996) to gauge traumatic distress in relation to the most prominent forced clearance described in the interview. The interview concluded with a participant debrief, which included a voluntary brief positive mood exercise (Holmes et al., 2009).

### Data analysis

Reflexive TA (Braun & Clarke, 2006, 2021) follows a six‐phase process, consisting of: (1) data familiarisation, (2) coding, (3) initial theme generation, (4) developing and reviewing themes, (5) refining, defining and naming themes and (6) writing up. Each interview was transcribed by the Lead Researcher, which facilitated data familiarisation, and each transcript was read through for further familiarisation. Interview data was coded using NVivo 14, from interview one through to nine. Coding was then revisited from interview nine through to one. Initial coding was completed at the semantic level, and revisiting the data progressed coding to the latent level. Notes were made throughout the coding regarding potential themes and initial themes created. Themes were refined through returning to the code set to confirm themes were capturing original codes.

Reflexive TA methodology provides a flexible orientation towards the data set. An inductive approach was taken due to the lack of pre‐existing theory and research related to forced clearances. Reflexive TA also allows for meanings to be derived from the data. The “reflexive” aspect of TA ensures that researcher experiences, assumptions and biases are reflected upon and their influence upon the research is acknowledged.

### Quality control

To establish methodological rigour, Braun and Clarke (2022) was followed, which offers 10 recommendations for how to avoid “common problems” in Reflexive TA, such as ensuring themes are meaning‐based rather than representing a topic summary. Other strategies to ensure rigour were consistent with Ahmed's (2024) principles of trustworthy qualitative research. For example, credibility was achieved by triangulating data across qualitative and quantitative methods; increasing transferability through the use of a homogenous sample; pre‐registration of the project and being transparent about data collection methods such as the interview transcript created dependability; and confirmability through the use of reflexivity.

### Epistemological statement

A critical realist (Bhaskar, 2008) stance was taken in relation to the research, which posits that truths are based in situational contexts involving individual, social and cultural factors. The data gathered is an interpretation of events, with different people deriving different realities from the same experience. The role of the researcher was to capture the nature of these subjective experiences.

### Reflexivity statement

The research was supervised by two Clinical Psychologists (JDG and LW), both of whom are accredited by the British Association for Behavioural and Cognitive Psychotherapies (BABCP). Therefore, a CBT orientation may have had a dominant influence upon the research. The Lead Researcher (HP) attended regular supervision as a method of reflexivity, in which both co‐authors held these influences in mind and acknowledged these influences throughout. The Lead Researcher also kept a reflective journal (Ortlipp, 2008) noting down observations and reflections throughout the research process, and especially in relation to recruitment and participant interviews.

## RESULTS

### Sample characteristics

Nine participants were interviewed. The age of participants ranged from 33 years and 4 months to 74 years and 4 months (*M* = 58 years and 9 months) and there were seven females and two males. Participants' ethnicity was White British (*n* = 4), Mixed White (*n* = 2), British Caribbean (*n* = 1), White Welsh (*n* = 1) and White Scottish (*n* = 1). Participants described experiences of clearances which were completed by organisations and family members and there was a wide range in the time since the clearances occurred (see Table 1). All participants met DSM‐5 (APA, 2013) criteria for HD; a co‐author (JDG) experienced in using the SIHD reviewed each interview to confirm the presence of HD and there was 100% agreement. In addition, all participants scored over the cut off (≥40) on the SI‐R indicating clinically significant hoarding and seven out of nine participants scored above the cut‐off (≥33) on the IES‐R for clinically significant symptoms (see Table 1).

**TABLE 1:** Characteristics.

	Approximate length of hoarding difficulties	SI‐R total score	Who conducted the clearance?	Approximate length of time since clearance	IES‐R total score
Participant 1	3 years	65	Housing association	3 years	59
Participant 2	30 years	63	Housing association	2 years	25
Participant 3	46 years	52	Family member	8 years	54
Participant 4	50 years	75	Council	7.5 weeks	61
Participant 5	55 years	56	Family member	6 years	0
Participant 6	Since childhood	70	Family member	37 years	51
Participant 7	34 years	59	Family member	32 years	54
Participant 8	15 years	83	Council	4 years	84
Participant 9	30 years	43	House repossession	10 years	59

### Experiences of forced clearances

Three themes and six subthemes were created from the data which are summarised in Table 2.

**TABLE 2:** Themes and subthemes from the experiences of forced clearances.

Themes	Subthemes
1. Power Imbalance	1.1 Criminalisation of Mental Health Difficulties 1.2 Clearance as the Lesser of Two Evils
2. The Trauma of Clearance	2.1 Losing Belongings is Like Losing Myself 2.2 ‘Trauma on top of Trauma’
3. Picking Up the Pieces	3.1 Clearance Weighs Heavily on the Mind 3.2 Replacing the Shield

#### Power Imbalance

This theme reflects the Power Imbalance between the individual with HD and those imposing the clearance. This was especially inherent in clearances completed by authorities, rather than family members, given they have statutory powers to clear properties such as under Section 83 of the Public Health Act (1936).

##### Criminalisation of mental health difficulties

This subtheme describes the experience of being made to feel like a criminal due to hoarding. Participant 4 described the correspondence they received:

…it was a standardised letter which said that if you don't clear it, this is antisocial behaviour, we'll force a Community Protection Notice and you will be fined…and taken to court…I didn't know at this point that police powers had been handed out to civil organisations.

They went on to say “…it felt as though I was a criminal because of this anti‐social behaviour, because of this police stamp on the letter as well”. This describes the use of punishment rather than support for these matters, and signalled a shift in identity for the participant of being a law‐abiding citizen to someone who potentially commits criminal offences. This may have made it very difficult for the individual to trust authorities, consistent with the literature describing the importance of PWHD needing to have trust (Brown & Pain, 2014).

##### Clearance as the lesser of two evils

Participants described clearance as representing the best of two unappealing options. For example, Participant 2 outlined:

If I didn't agree to the house clearance, the housing association would have forced it on me anyway…and charged me for it…it would have taken years and years to pay off cause I'm on benefits…technically I agreed to it, but I had no choice.

Further, Participant 3 described potential future consequences of not maintaining the results of the clearance “Well, I suppose…it reminds me that I must keep it clear or I will lose my flat”, having previously been told that if they do not clear it then their flat will be taken from them.

Agreeing to a clearance intervention could be described as an illusion of choice, given the options are posed as a choice, but offer no feasible alternative to the individual. Participant 2 further described: “…it had to happen, I had no control over it, you know even if I'd said no it would have been forced on me which would probably have been even more traumatic, you know what I mean?”

#### The Trauma of Clearance

This theme describes the traumatic nature of forced clearance, both in the context of previous trauma experiences commonly reported across participants and through aspects of the clearance which contributed to the distress of the experience.

##### Losing belongings is like losing myself

Participants described the loss of belongings as a visceral experience, making references to losing part of themselves and using physical descriptors. Participant 6 described “…losing it is, it feels like my own personal death. It's horrific…it's horrible because it feels like they have, part of my soul is with them and I don't know what's happened to it”. This captures the enmeshment between people and their belongings. Participant 6 also described their disbelief in still existing after the loss of their belongings:

It's a really similar feeling of total, complete loss, but it's not just that I've lost something. It's, I've lost part of myself and how can I continue breathing and living? It's a really weird feeling and kind of a shock like I am still here, but how?

Participant 7 reported: “I was gonna say a word that I haven't heard myself say before, but it's like a crack in your heart…when all your stuff's thrown away”. Participant 1 also described what the loss of their belongings represented: “It's something to, to really think about because I think losing those things, you know how you got them and you know what they mean to you, the sort of attachment”.

Increased arousal and strong emotions in the context of matching triggers was an additional feature, Participant 6 said:

And it's a weird one, like, cause when my bag got stolen and it had, it was a reminder that all of my most precious things are gone and the whole like, the whole body feels, I don't know, how do you explain it?

Participant 7 also described their experience with discarding: “And it's so hard, you feel your whole body wracked with pain doing it and it's like, and usually often I cry, it's that painful, picking up the next piece of paper…”.

##### Trauma on top of trauma

This subtheme captures both the nature of the clearance being traumatic on top of the clearance itself, but also that a clearance was another traumatic event on top of a history of trauma. For some participants, the experience of clearance was already considered traumatic, but made worse by belongings being stolen in the process, as was the case for Participant 2: “…there was stuff gone through, you know stuff had been moved about, chucked about, looking for the good stuff to take, basically…So that was trauma on top of trauma”. The role of trauma was for some, part of their history and identity and participants described how these vulnerabilities were not taken into account. This was described by Participant 8: “…let's say I was damaged goods to start with…But, they didn't take anything into consideration…about my mental state…”. This was also supported by Participant 4:

And so in those aspects, it's worse because it's confirmed to me how, umm, ah it's worse than insensitive, but how, if you like, violent, brutal, how, how, if you like they're prepared to take the law into their own hands and administer it at will without any consideration to the impact or to managing the situation as a whole, to managing the person.

Participant 6 described the loss of their belongings in the context of a previous bereavement, stating “And one of those people had died, and that was the only memory I had of that person, and that memory was of that person, and now it's washed and gone, and that memory is gone”.

For Participant 8, the research interview itself allowed them to realise that they were experiencing similar symptoms in the context of the clearance as they had compared to PTSD symptoms from a separate event: “Yeah, but it does make us wonder, something I hadn't realised, the post‐traumatic stress, the symptoms are the same…It explains an awful lot”.

#### Picking Up the Pieces

This theme reports how participants responded in the aftermath of the clearance and how they appraised what happened. Both cognitive and behavioural strategies for managing the clearance are identified.

##### Clearance weighs heavily on the mind

This subtheme describes participants' negative appraisals given to the event and its sequelae, and the ruminative thinking about their lost belongings. For example, Participant 7 described: “…so if somebody is forcing me to [discard possessions] then I feel violated, it's like being abused”, and Participant 6 explained how the clearance affected their relationship with their parents: “Yes, I didn't trust them. I didn't trust them”.

Participant 9 described their regret at which belongings they chose to keep: “It was mortifying, and obviously like, this, done it the last time as well, picked up stupid things…You just don't know what to pick up, you know you…Yeah. Terrible. Yeah, it still hurts”. Similarly, Participant 6 stated: “It's just constant anxiety. Especially the things that I wanted to keep that have been disappeared because of the moving and it's always in my head, “where are they? Where are they?”. I wish I had them. Yeah, it's constant”. Participant 3 detailed how they missed spending time with their belongings at a storage unit which could be perceived as a loss of meaningful occupation for them:

Well unhappy because I didn't, couldn't remember everything that was down in the storage unit, but I used to go down and I missed that going down there because I got exercise as well because if it was a nice day I'd walk down…I'd take some lunch with me and a drink, and I quite enjoyed it and I enjoyed talking to the ladies that worked in the office…And it helped the depression as well.

##### Replacing the shield

This subtheme captures the function of hoarding for the individual, the vulnerability that the clearance exposes for them, and the need to reinstate a sense of safety.

Participant 5 described: “Yeah, it's a weird feeling that, I don't like them [possessions] leaving me because they make me feel safe and I like to have them around me”, and how they instinctively tried to recover their belongings:

I was really cross…I even went out and drove round where she might have put the stuff…so I even went and looked in bins to see if I could find my things…Well, I thought, what am I doing? This is ridiculous. Why, why would I do this? It felt important to me at the time.

Participant 6 reported increased difficulty parting with possessions as a direct result of losing their belongings:

…because of the trauma of that situation, I started hoarding even more…There's a deeper fear, it's like I need more to hang on to this. It's a really weird theory actually, like that happened, now I have to do it twice as much…Somehow that would be safer, like get more of it to stop that from happening.

Participant 7 also explained why they acquired more after losing their belongings: “Umm, identity…This is who I am. You know, this is me…self‐expression, yeah. It's all, umm, it's well deeper than that, it's a fight for survival, there's something about, “I am here”, you know?”. This would suggest that clearance is counterproductive, offering only a short‐term solution to managing clutter, and in fact could worsen difficulties with hoarding by driving individuals to acquire more in an effort to prevent future loss and to re‐claim their sense of identity.

## DISCUSSION

This thematic analysis directly explored the experience of forced clearances in PWHD, resulting in themes of Power Imbalance (subthemes: Criminalisation of Mental Health Difficulties; Clearance as the Lesser of Two Evils); The Trauma of Clearance (subthemes: Losing Belongings is Like Losing Myself; ‘Trauma on top of Trauma’); and Picking Up the Pieces (subthemes: Clearance Weighs Heavily on the Mind; Replacing the Shield). Overall, the findings describe forced clearance as an experience which is inherently subject to a Power Imbalance resulting in individuals with HD experiencing a lack of choice and control. Further, the results indicate that a forced clearance is a traumatic experience that can contribute to refuge being sought in belongings and furthering the need to hoard, demonstrating the counterproductive nature of clearance.

This distress could be theoretically underpinned by clearance as a violation of the individual's secure site, or home (Kellett, 2007), explaining the drive reported by participants to recover or replace their belongings in an attempt to restore security of their site. This response was observed in the theme ‘Picking Up the Pieces’ and the subtheme of ‘Replacing the Shield’. The findings therefore support that a forced clearance would be expected to worsen hoarding difficulties, with participants reporting a drive to acquire more belongings to prevent future clearance from happening again. Evidence suggests that PWHD experience a feeling of emptiness upon the loss of belongings (Kings et al., 2021), which could further explain the need to fill this void. A further reason that these experiences were so distressing may in part be due to how the clearance was completed. Participants reported financial consequences for not engaging with the intervention (captured by the theme of ‘Power Imbalance’ and the subtheme of ‘Clearance as the Lesser of Two Evils’) and last‐minute changes to the date of the clearance and misunderstanding about the area to be cleared.

The intense experience of loss that participants reported in the subtheme Losing Belongings is Like Losing Myself could be due to the perception of threat to self. The subjective descriptions of trauma are complemented by the evidence that such experiences remain traumatic many years afterwards, as suggested by the IES‐R (Weiss & Marmar, 1996) scores. However, it is important to note that a low IES‐R score does not mean that a forced clearance was not experienced as traumatic by an individual. For example, Participant 5, who experienced a negative forced clearance from a family member, recognised that the action was well intended and did not endorse any items on the IES‐R (Weiss & Marmar, 1996). Despite PWHD reporting an increased frequency of traumatic events and a higher incidence of childhood trauma compared to people with OCD, they do not typically present with elevated rates of PTSD (Frost et al., 2011). This may be explained by the assessment of PTSD in the aforementioned study asking participants to recall an “…extremely stressful, life‐threatening, or traumatic event such as serious physical injury, rape, assault or combat…”, which may not necessarily prompt recall of loss of belongings through a forced clearance as an example. The current study therefore provides some evidence to suggest that if individuals are oriented to report on a forced clearance as a traumatic event, they may report symptoms consistent with a PTSD presentation, although this would not meet PTSD diagnostic criteria (APA, 2013). This would also be consistent with other types of traumas that can exist in the absence of criterion A for PTSD (APA, 2013), for example, social trauma such as bullying (Hardarson et al., 2023) and supports the argument for a broader, evolutionary definition of trauma (Bjornsson et al., 2020). The findings are consistent with cognitive models of PTSD (e.g. Ehlers & Clark, 2000). Post‐traumatic appraisals were described in the subtheme of Clearance Weighs Heavily on the Mind, with the traumatic appraisal given to the event being indicative of both the likelihood of a PTSD reaction and symptom severity (Beierl et al., 2020; Ehlers & Clark, 2000). Strategies intended to control threat symptoms were also seen in participants including ruminating on the loss of belongings and what could have been done differently which also maintains the PTSD response (Ehlers & Clark, 2000). Future research would benefit from further exploring the negative appraisals given to the clearance experience, as this may identify key cognitions related to the self, others and future, which are maintaining factors in PTSD (Ehlers & Clark, 2000).

There are several clinical implications of the current study. A forced intervention should only be an intervention of last resort. There may be occasions when such interventions are required by agencies with statutory responsibilities, but if this is the case, then PWHD are likely to benefit from intervention being aligned with a trauma‐informed approach (SAMHSA, 2014), which aims to prevent re‐traumatisation, to work collaboratively with individuals and to build trust and offer choice. It would be beneficial for clinicians working therapeutically with HD to assess for previous experiences of clearance and to understand the potential impact that this may have on engagement with help and the therapeutic relationship. It would also be important for multi‐disciplinary team members, such as housing officers, social workers and environmental health, to consider these findings so that their practice can also be adapted, especially given the wide range of disciplines involved with HD (Holden et al., 2019; Porter & Hanson, 2022). With the high rates of trauma in PWHD (Landau et al., 2011), there are several adaptations which could prove to be simple but effective in helping the individual to have more control in the situation and to prevent the layers of trauma that were described in the current study. The current study would imply other strategies could include agreeing the areas to be cleared in advance and ensuring both parties understand this, making every attempt not to change the agreed date and time of intervention, and offering post‐clearance support. It could also be helpful for PWHD to be offered an advocate to liaise on their behalf, given the difficulties reported by participants in making contact with authorities, as well as being offered evidence‐based therapy for traumatic experiences resulting from a forced clearance, consistent with participants recommending access to trauma therapy. Further participant recommendations on what improved support could look like are available in the supplementary material.

Strengths of the current study include that HD was established using the DSM‐5 (APA, 2013) diagnostic interview, as opposed to self‐identification of HD, which therefore helped to establish a homogenous group of participants. Although generalisability is not a given outcome of qualitative research (Leung, 2015), the homogeneity in this study does allow for transferable conclusions about HD to be drawn, making a meaningful contribution to the field of HD research. Furthermore, the study also benefited from using a semi‐structured interview method to explore experiences in a flexible way, but psychological measures were also used to quantitatively establish the severity of hoarding and the traumatic nature of the clearance experience. It is worth noting that the current study is the first to recruit PWHD to a study about experiences of forced clearances; given the specificity of the group and the phenomena under investigation, the study is considered to have significant information power (Malterud et al., 2016).

In terms of limitations, we know that PWHD are socially isolated (Davidson et al., 2020) and therefore the current study may not have captured the experiences of those who are more vulnerable. For example, the study was unlikely to have reached individuals who may have experienced clearance interventions but do not utilise hoarding support groups, which was the primary recruitment method. The sample represents those who felt able to talk about their experience, but did not capture the experiences of those who felt it was too distressing to talk about in a research context, with differences between research and community HD samples highlighted by Woody et al. (2020). The current study understood the findings from the perspective of CBT trauma models due to the important role that these have made in understanding and treating the consequences of traumatic events in areas such as PTSD (e.g. Brewin et al., 2010) and Complex PTSD (e.g. Hyland et al., 2023). Alternative frameworks such as attachment theory (Ainsworth & Bowlby, 1991) have also been drawn upon to understand the developmental context in which hoarding problems may emerge in relation to early life experiences, such as traumatic events (see Chia et al., 2021). Completion of the IES‐R was subject to retrospective recall of the traumatic event and symptoms over the last month; for some participants, the event took place many years previously. Future research could incorporate ecological momentary assessment (Shiffman et al., 2008) to increase accuracy of reporting of data. However, this limitation is not unique to this study and would be a feature of any research using retrospective self‐report measures.

## CONCLUSION

The study provides the first recorded accounts of PWHD's experiences of forced clearances, with the results conveying the profound, negative and long‐term impact of this action on them. It is important for future research to explore how common forced clearances are given how little is currently known about this practice, as well as establishing the various impacts, including costs, both financially to services and to the psychological wellbeing of individuals experiencing it. It would also be important to explore the experiences of professionals who have been involved in forced clearance interventions, which may help to consider how organisational goals can be met in such a way that supports the individual.

## AUTHOR CONTRIBUTIONS

**Hannah Parker:** Conceptualization; methodology; data curation; formal analysis; writing – original draft; writing – review and editing. **Louise Waddington:** Conceptualization; methodology; supervision; formal analysis; writing – review and editing. **James D. Gregory:** Conceptualization; methodology; supervision; formal analysis; writing – review and editing.

## CONFLICT OF INTEREST STATEMENT

None of the authors have a conflict of interest to disclose.

## Supporting information


Data S1:

## Data Availability

Research data are not shared.
