# Supplementary material for: Exploring experiences of forced clearances in people with hoarding difficulties
Source: Psychol Psychother. 2026 Jun 7;99(4):1442–56. doi: 10.1111/papt.70082 (PMC13749239; doi:10.1111/papt.70082)
Supplement: Supplementary file 1 [file PAPT-99-1442-s001.docx]

| Recommendation | Participant Quotation |
| --- | --- |
| HD Requires Early Intervention | “But I don’t think a lot of housing associations have that, you know, “well this is mild hoarding but...maybe we should address it now before it becomes...extreme hoarding”, you know?” (Participant 2)  “To a certain extent if you had someone keeping an eye on you before it gets, maybe if she’d come last September...it wouldn’t be as bad as it is now, if you know what I mean? Maybe if she’d said something then it would have stopped it getting even worse” (Participant 2)  “Well, I wonder if perhaps my [relative] had got some help for me, I wonder if that might have made a difference…well it’s a pity people don’t notice...when people are hoarding and maybe they could get help sooner rather than later” (Participant 3) |
| Services Need to Recognise HD as a Mental Health Problem | “While hoarding is recognised as a mental disorder by the NHS and the World Health Organisation, this psychologist did not, just didn’t take it seriously at all…They never, she refused to ever meet me, she decided I should have occupational therapy, I said from the start, I doubt occupational therapy on its own will work...without sort of psychological input...and I just wasn’t listened to” (Participant 2)  “No, but it's recognised as an illness now...or a disability or something so” (Participant 3) |
| “See the Person and Not the Stuff” | “…doing some work with (name), made me realise that the most important thing is to see the person and not the stuff” (Participant 4)  “Umm, sitting, listen to them with a problem, before you pick a bag up” (Participant 8)  “People really need to like, understand, not just judge straight off, you know? Really, that's really big one, instead of just, you know, coming straight in, judging, you need to know the back story because, you know, they've all got the back story” (Participant 9) |
| Importance of Building Trust | “But if you do know those items are there then then you need to discuss it first before, before that somebody decides to take them or you agreed for someone to take them” (Participant 5)  “The collaboration but also not just that, you have to gain some trust and to have some kind of, uh, ability to understand that person and to feel comfortable with that person as well, that they're not, that they're like, that they might not be the right person, but it could be someone else that might be the right person” (Participant 5) |
| Know Your Rights | “But I think that is something really, really fundamentally important to focus on is to look at what are your rights, who can you talk to? Well, what are the important phone numbers?” (Participant 4) |
| Have Patience and Work Flexibly | “Don't pressure people, don’t pressure people into it. That is a horrible one” (Participant 6)  “If there's a timeline and they're not making it, give them another timeline, like be gentle with these people cause...it's really important. Have a helping hand, say “we're here to help you to clear it out” rather than “we're chucking your stuff” because the counter to that, let's say, oh, all their stuff, just gets chucked or it's done too quickly...it's not gonna be like it's just gonna go away…what you're going to do then next time they have a place is gonna be worse…It'll be a bigger problem down the line” (Participant 6)  “But it was something (de-clutterer) said the other day, one thing at a time...one thing a day. And that, to me was brilliant” (Participant 8) |
| Start a Conversation | “…have a conversation with, to help them to put it down because one of the biggest things is if people don't go to the doctor and they don't go with some solid information, they won't get the help, and it needs to be flagged up more and more, for people to get the right type of help” (Participant 4)  “Talk to the people…Try and get them help from the proper place, a proper person…I know that some people are ideal for clearing. (name) is…She was a little darling. I had one other lady, they called her (name)…And she was very good” (Participant 8) |
| Offer Trauma Therapy | “Offer trauma therapy as well, that is specifically towards trauma…” (Participant 6)  “Access to counselling, PTSD and trauma support” (Participant 7) |
| Offer Practical Help for Sorting and Discarding | “Give alternatives. So for example, if they're able to, let's say it's an association or local authority and they need to move someone on who's got loads of stuff…Offer them like a warehouse that they can put their stuff in, somewhere they can direct their stuff, otherwise they gonna be too much stress to be able to even function…” (Participant 6)  “Practical help with selling things that hoarders are ready to let go but need recognition of the value of and the income from to bring the value of the items into present day use and alleviate their poverty” (Participant 7)  “Access to free loans of sorting tables, storage boxes and filing trolleys” (Participant 7) |
